# Simultaneous Thrombosis of Two Drug-Eluting Stents After Discontinuation of Dual Antiplatelet Therapy for a Day

**DOI:** 10.4021/cr242e

**Published:** 2012-11-20

**Authors:** Hung Yi Chen

**Keywords:** Subacute stent thrombosis, Drug-eluting stent, Dual antiplatelet therapy

## Abstract

Stent thrombosis is rare but can lead to potential severe consequence. The incidence is relative higher in drug-eluting sent than bare-metal stent implantation. Dual antiplatelet therapy is the major treatment to avoid early and late stent thrombosis. Simultaneous two stents thrombosis is rare. Although mechanical or/and procedure factors may predispose to stent thrombosis occurred, simultaneous two stents thrombosis implies possibly ineffective antiplatelet therapy. We report a case with simultaneous two stent thrombosis and complicated with cardiogenic shock after lost antiplatelet therapy for one day. We try to emphasize to properly educate patients about the importance of continuous drug use to avoid catastrophic tragedy.

## Introduction

Stent thrombosis is a challenging problem that can lead to serious clinical consequence. The phenomenon most commonly occurs in the first month after stent implantation. In addition to patient characteristics or procedure factors, inadequate dual antiplatelet therapy is the main cause. And premature discontinuation of dual antiplatelet remains the most powerful predictor for stent thrombosis. We report a case with simultaneous two stents thrombosis after lost antiplatelet therapy for one day. Without the information about inhibition of platelet activation, we do not know the impact of discontinuation of therapy for one day. However, we try to emphasize to properly educate patients the importance of drug use and the potential severe consequences of antiplatelet therapy cessation.

## Case Report

**Figure 1:**
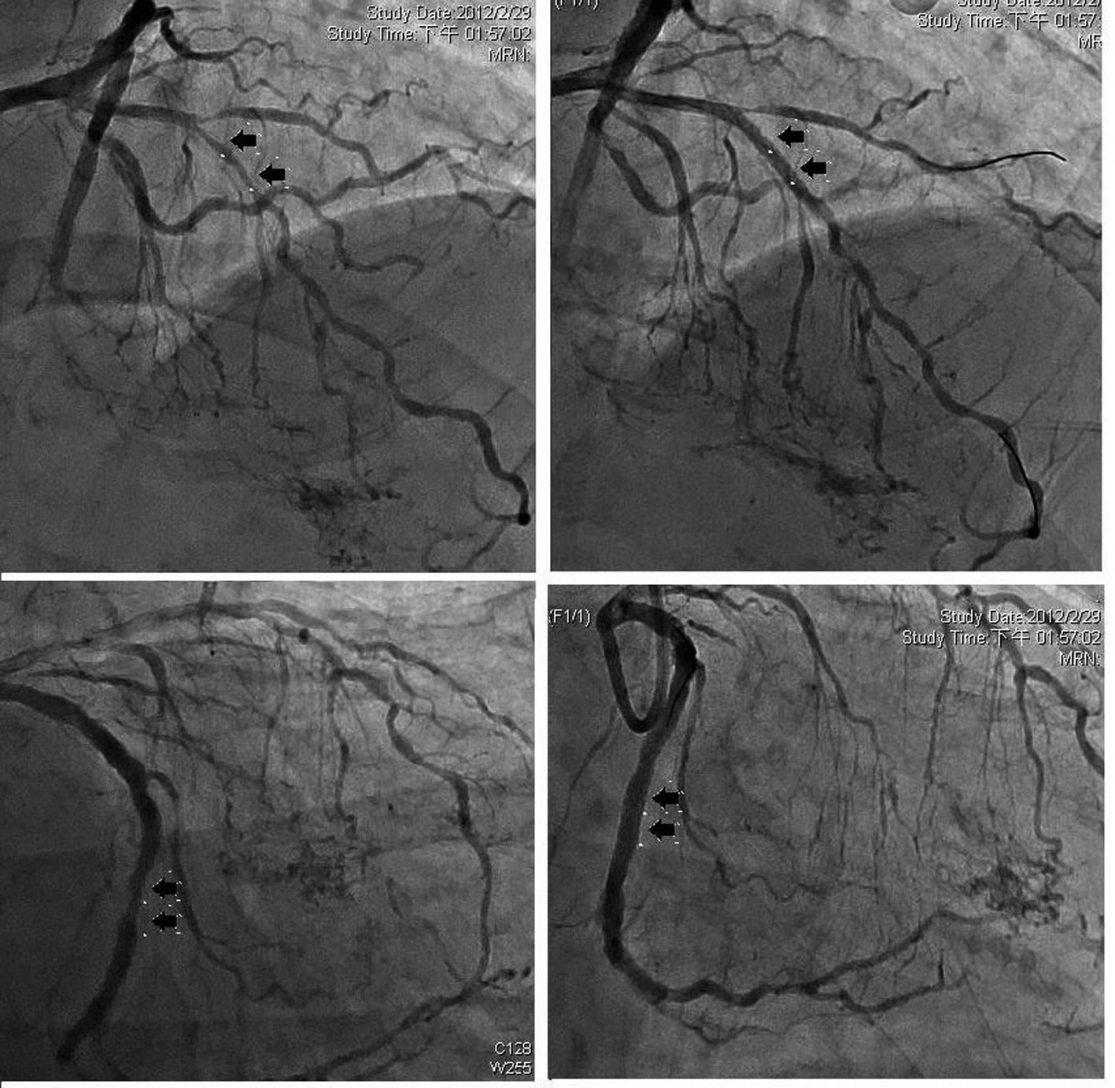
Left coronary angiography showed left anterior descending (upper panels) and left circumflex artery (lower panels), before (left) and after (right) stent implantation.

A 77-year-old man is a victim of hypertension and diabetes under insulin control at our outpatient clinic. He complained of chest tightness and dyspnea on exertion for a period. He had a history of tobacco used but quit for decades. Echocardiography was performed on 2011 September and the result showed normal left ventricular function without regional wall motion abnormality. Due to frequent chest tightness complained on exertion and the symptom exacerbated. He visited our emergency department for help. At emergency department, CXR showed cardiomegaly. Cardiac enzyme was checked and it was within normal limit. Under the impression of coronary artery disease with angina, he was treated with dual antiplatelet therapy (aspirin 100 mg/day, clopidogrel loading 300 mg, then 75 mg/day) at emergency department and catheterization was suggested after admission. Three days later, catheterization was done and coronary angiography demonstrated left anterior descending artery (LAD) with critical lesion and left circumflex (LCX) atherosclerotic lesion. Angioplasty was performed and two drug-eluting stents (BIOMATRIX 3.0 × 18 and 2.5 × 18) were deployed on LAD and LCX, respectively, with clopidogrel loading 300 mg (Fig. 1). The hospital course was uneventful and he was discharged on aspirin and clopidogrel on the following day.

**Figure 2:**
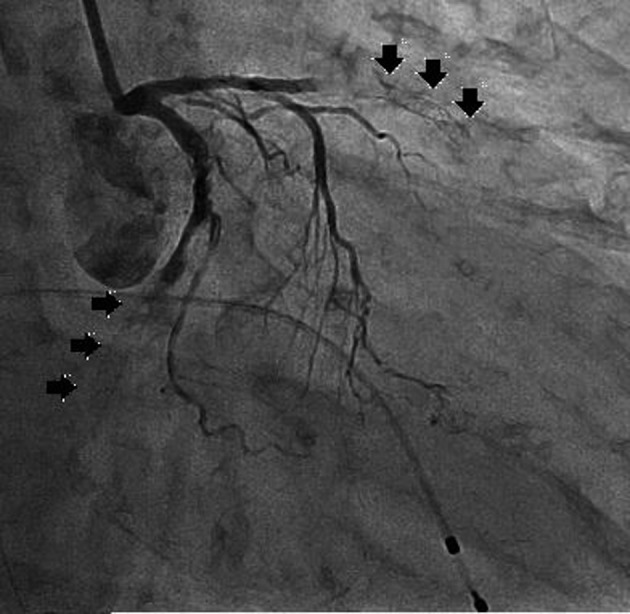
Left coronary angiography showed total occlusion at the proximal edge of the drug-eluting stents implanted in the mid anterior descending artery and mid left circumflex artery.

He was transferred to emergency room again at the noon on the next day after discharge, and he complained of severe acute onset chest pain with diaphoresis. On admission, his blood pressure was 150/96 mmHg, and pulse beats 110/min. His electrocardiography (ECG) showed ST elevation in leads V1-V5. Over the next 10 minutes, his blood pressure dropped to 65/48 mmHg with complete atrioventricular block by ECG monitor, and he was transferred immediately for emergency coronary angiography. After implantation of a temporal pacemaker, coronary angiography revealed totally occluded mid LAD and mid LCX at the same time, the site over the prior stents (Fig. 2). Balloon angioplasty restored Thrombolysis in Myocardial Infarction (TIMI) 2 flow in both sites. Because of low left ventricular ejection fraction and a large thrombus burden by SETMI secondary to stents thrombosis, re-occlusion developed later resulted in failure from primary PCI.

## Discussion

Definition by the Academic Research Consortium definite definition, acute stent thrombosis was categorized as occurrence within the first 24 hours after the index procedure, and subacute as occurrence from 24 hours to 30 days. Acute and subacute stent thromboses together were categorized as early stent thrombosis. Late stent thrombosis was categorized as 31 days to 1 year post procedure, and very late more than 1 year after procedure [1].

Stent thrombosis is a challenging problem that can lead to serious clinical consequence. Its pathophysiology is not completely known, and there are several causes suggested such as incomplete stent endothelization, presence of polymers and late incomplete stent apposition. The incidence is relative higher in drug-eluting sent in early and late stent thrombosis comparing with bare-metal stent [2]. The possible predictors of stent thrombosis can be categorized as underlying patient, lesion and procedure characteristics. In general, stent thrombosis occurs more frequently in complex patients with complex lesions as diabetes, chronic kidney disease, acute coronary syndrome, multiple stents implantation, bifurcation lesions and chronic total occlusion [3-5]. When acute stent thrombosis development, it is commonly related to procedure as incomplete stent expansion, edge dissection, stent malapposition, strut fracture and reduced TIMI flow [3, 6-8]. Stent thrombosis most commonly occurs in the first month after stent implantation. Under continuous dual antiplatelet therapy, the average occurrence of subacute thrombosis in drug-eluting stent is 0.5-2% [9-11]. However, discontinuation of dual antiplatelet therapy was the most powerful predictor of stent thrombosis during the first 6 months following stent implantation in cohort studies over 3,000 patients [2]. Even in late stent thrombosis, ineffective dual antiplatelet therapy remained the common reason [12].

As to the time duration between discontinuation of dual antiplatelet therapy and subacute stent thrombosis, it is difficult to detailed assess because antiplatelet medications are routinely used after stent implantation in present time. There was recent evidence conducted by Heestermans et al They investigated early coronary stent thrombosis in 5,842 patients with ST segment elevation myocardial infarction (STEMI) and received primary percutaneous coronary intervention. In their data, there were 18.5% patients with subacute stent thrombosis without clopidogrel maintenance therapy, and clopidogrel was stopped at an average of 6.7 ± 5.8 days before the development of subacute stent thrombosis [13].

Simultaneous two stents thrombosis was extremely rare. In our case, although mechanical or/and procedure factors may predispose to stent thrombosis, both two stents thrombosis suggested failure of antiplatelet therapy. The reason for stent thrombosis in the case can be either clopidogrel resistance or discontinuation of clopidogrel. Because information on inhibition of platelet activation was not available in this case, we do not know the impact of losing antiplatelet therapy for one day on stent thrombosis. However, the case strongly suggested that it should be emphasized to ensure patients to be properly educated about the importance of drug use and the potential severe consequences of antiplatelet therapy cessation.
